# Biomechanically optimized 3D-Printed titanium prostheses with stiffener arrangement for critical femoral diaphyseal defects: early weight-bearing capacity and combat readiness validated through integrated biomechanical-FEA approach

**DOI:** 10.3389/fbioe.2025.1642787

**Published:** 2025-09-17

**Authors:** Guo-Sen Li, Hao Li, Da Liu, Rui Yi, Yi Cui, Hong-Da Lao, Xiao-Yang Nie, Min Zhao, Cheng-Fei Du, Yong-Qing Xu, Jiang-Jun Zhou

**Affiliations:** ^1^ Department of Orthopaedics, The 908th Hospital of Joint Logistic Support Force, Nanchang, Jiangxi, China; ^2^ Third Clinical Medical College of Nanchang University, Jiangxi Medical College, Nanchang University, Nanchang, Jiangxi, China; ^3^ The General Hospital of Western Theater Command, Chengdu, Sichuan, China; ^4^ The Seventh Medical Center, Chinese PLA General Hospital, Beijing, China; ^5^ Institute of Orthopedic Trauma, 920th Hospital of Chinese PLA Joint Logistics Support Force, Kunming, Yunnan, China; ^6^ Department of Orthopaedics, The People’s Hospital of Yingtan City, Yingtan, Jiangxi, China; ^7^ Tianjin Key Laboratory for Advanced Mechatronic System Design and Intelligent Control, School of Mechanical Engineering, Tianjin University of Technology, Tianjin, China

**Keywords:** 3D-printed prosthesis, critical bone defect, finite element analysis, biomechanical compatibility, military trauma, stiffener, titanium alloy

## Abstract

**Introduction:**

Critical femoral diaphyseal defects exceeding 3 cm present significant challenges in trauma and military orthopedics, particularly in blast injury scenarios requiring rapid rehabilitation.

**Methods:**

The purpose of this experiment was to evaluate the biomechanical *in vitro* performance of two personalized prostheses (Groups A and B) designed explicitly for critical femoral diaphyseal defects through integrated biomechanical testing and finite element analysis (FEA). Using fourth-generation composite femurs simulating 10 cm defects (n = 16), we compared axial compression, torsion, four-point bending stiffness, and cyclic fatigue performance against intact bones (Group D) and diaphyseal fractures without defects (Group C).

**Results:**

Key findings demonstrate comparable compressive stiffness between prostheses groups (Group A: 764.12±112.63 N/mm; Group B: 693.63±136.31 N/mm) and intact femurs (808.59±18.1 N/mm, p>0.05). The torsional stiffness is comparable between prostheses groups (Group A: 2.28±0.15 Nm/°; Group B: 2.18±0.22 Nm/°) versus diaphyseal fractures without defects (2.01±0.19 Nm/°). The stiffness results comply with mobilization requirements. FEA revealed maximum von Mises stresses in prosthesis fixation systems below the yield strength of Ti6Al4V, with digital image correlation validating the stress distribution patterns. The porous scaffold design achieved optimal modulus (1,132.85 MPa) between cortical and cancellous bone, reducing the “stress shielding” effect. Both prostheses endured 1800 N cyclic loading (100,000 cycles ≈, 13.3 years of physiological use) without structural failure.

**Discussion:**

These customized prostheses address critical military medical needs by enabling immediate weight-bearing, reducing surgical complexity compared to bone transport techniques, and maintaining long-term mechanical integrity. The stiffener design philosophy and additive manufacturing flexibility provide adaptable solutions for complex combat-related trauma, significantly advancing early functional recovery in resource-constrained environments.

## 1 Introduction

Bone is one of nature’s finest “composites” with great self-healing ability and excellent biomechanical properties. However, these are particularly weak in addressing severe and significant segment defects. When the length of the defect reaches 1.5–3.0 cm or is 1.5 times the diameter of the diaphysis, it exceeds the limit of natural bone healing and requires further surgical intervention; this type of defect is known as a “critical bone defect.” Bone defects may result from several etiologies, including high-energy trauma accompanied by soft tissue and periosteal separation (notably high-grade open tibial and femur fractures), blast injuries, infections requiring extensive debridement, and tumor resections (Eckstein et al., 2002). Orthopedic surgeons conduct about 2 million bone grafting procedures annually to treat bone defects (Archunan and Petronis, 2021). Since the Russian Federation’s invasion of Ukraine commenced in February 2022, 90% of injuries sustained by Ukrainian soldiers affect the limbs, with approximately one-third being fractures of long bones (Lawry et al., 2025). According to a report on battle casualties from NATO coalition forces in Iraq and Afghanistan, explosive devices are the signature threat of current military operations, with blast injury being the primary mechanism of injury, significantly higher than gunshot injury (Hoencamp et al., 2014). Modern warfare has a high proportion of blast injuries, and high-intensity conflicts can lead to large numbers of injuries and saturation of medical resources, requiring advanced planning of treatment strategies (Mathieu et al., 2025).

For those facing severe bone defects, autograft is currently the gold standard. Autograft is limited in quantity, and excessive bone harvesting can cause complications in the supply area (Sun, Tang, and Zhang, 2024). Allograft and xenograft offer the advantage of having relatively diverse and abundant sources, such as cadavers or other mammals like bovine and porcine (Ferraz, 2023). However, allografts and xenografts may cause an immune rejection reaction and increase the risk of infectious diseases (Sohn and Oh, 2019). Preprocessing resulted in a loss of its osteoinductive ability. Artificial bone, such as calcium phosphate and calcium sulfate, has a similar composition to human bone inorganic salts. Artificial bone is suitable for filling bone defects, but its osteoinductive and long-term load-bearing ability performance is suboptimal. (durable bone support) Bone transport techniques can treat bone defects with the help of Ilizarov external fixators. Nevertheless, the long-term exposure of pin tracts renders this technique prone to infection (Hamiti et al., 2023). Magnesium alloy is considered a biodegradable metal material; however, its low yield strength (157MPa, 36–40 MPa for porous structure) makes it challenging to apply to weight-bearing bones like the femur (Liang et al., 2023; Chen and Chen, 2024). The *in vivo* dissolvable nature makes its strength for long-term prosthesization challenging to assess quantitatively (Witte et al., 2006). Its high biocompatibility also implies that it is more susceptible to degradation within the body. Tantalum prostheses have excellent mechanical properties, good biocompatibility, and the potential to promote angiogenesis, making them one of the ideal materials for the fabrication of bone prostheses; however, due to the difficulty of processing and high price, the clinical applications and evaluations of long-term use are limited (Cardarelli, 2001; Carraro and Bagno, 2023; Liu et al., 2025).

Ti6Al4V titanium alloy exhibits approximately 40% greater strength than traditional titanium alloys, while maintaining commendable toughness and ductility, making it suitable for intricate structures and high-strength applications. The alloy exhibits exceptional stability in diverse adverse settings, showcasing significant resistance to various chemicals and corrosive substances, thereby prolonging product longevity (Gong et al., 2025). The Ti6Al4V titanium alloy consistently performs well under cyclic stress, demonstrating resilience against fatigue failure (El Hassanin, Scherillo, et al.). This distinctive feature renders it exceptionally beneficial in high-frequency and high-load applications. 3D-printed titanium alloy (Ti6Al4V) products offer excellent anatomical shape matching, possess a higher yield strength (819–1,022 MPa), and allow for modulation of the elastic modulus through scaffolds porosity (Wang et al., 2024; Yang et al., 2024). Ti6Al4V is widely used in the manufacture of prosthetic medical devices due to its excellent biocompatibility and mechanical properties (Gong et al., 2025). The excellent biocompatibility and resistance to corrosion of titanium alloy ensure the long-term effectiveness of its support (Bose et al., 2018). The titanium alloy prosthesis provides immediate stability and facilitates recovery and early mobilization by eliminating the time required for bone formation and directly filling the bone deficiency, which is crucial in a battlefield environment (Palumbo et al., 2011; Zheng et al., 2019; Mathieu et al., 2025).

Relevant studies have found that a prosthesis porosity of more than 70% is favourable for bone growth and osseointegration; however, ensuring the yield strength of the prosthesis is difficult at this porosity (Jiao et al., 2023). In the field of engineering design, stiffener is commonly used to improve the strength and stability of metal parts, which can disperse stresses and reduce stress concentration, thus enhancing the durability of the overall structure. Therefore, we utilize the design of a crossing stiffener to balance the stiffness and porosity of the prosthesis.

Biomechanical experiments can help evaluate the mechanical properties of a prosthesis in a simulated physiological environment, such as strength, stiffness, stability, and durability, to ensure that the prosthesis can withstand the various loads generated by the body’s daily activities (Nasrin et al., 2024; Zimmerman et al., 2024). Femoral composite Sawbones were used to replace cadaver femurs due to their homogeneous mechanical properties, which can effectively reduce individual differences between samples and the bias of experimental data (Reed et al., 2013; Elfar et al., 2014). Digital image correlation (DIC) enables the acquisition of full-field strain and displacement measurements, which can be used to verify the accuracy of finite element analysis (FEA) and ensure the reliability of simulation results.

Through high-fidelity computational modeling, FEA provides quantitatively validated predictions of the maximum von Mises stress and stress distribution, outperforming traditional biomechanical testing in both spatial resolution and parametric control (Wang and Wu, 2023).

This study aims to evaluate the biomechanical properties and clinical feasibility of prostheses A and B through biomechanical testing and finite element analysis. Femoral composite Sawbones were used to simulate the femur in biomechanical experiments. This study aims to provide clinicians with a broader range of treatment options for femoral shaft fractures with Femoral Critical Diaphyseal Defects. Femoral shaft transverse fractures are more clinically manageable than Femoral Critical Diaphyseal Defects. Femoral shaft transverse fractures are fixed with double plates, which provide favorable biomechanical properties. Therefore, we used double plating of transverse fractures and an intact femur as a control to compare the extent to which the two prostheses we designed can mitigate the biomechanical effects of critical bone defects.

## 2 Materials and methods

**FIGURE 1:**
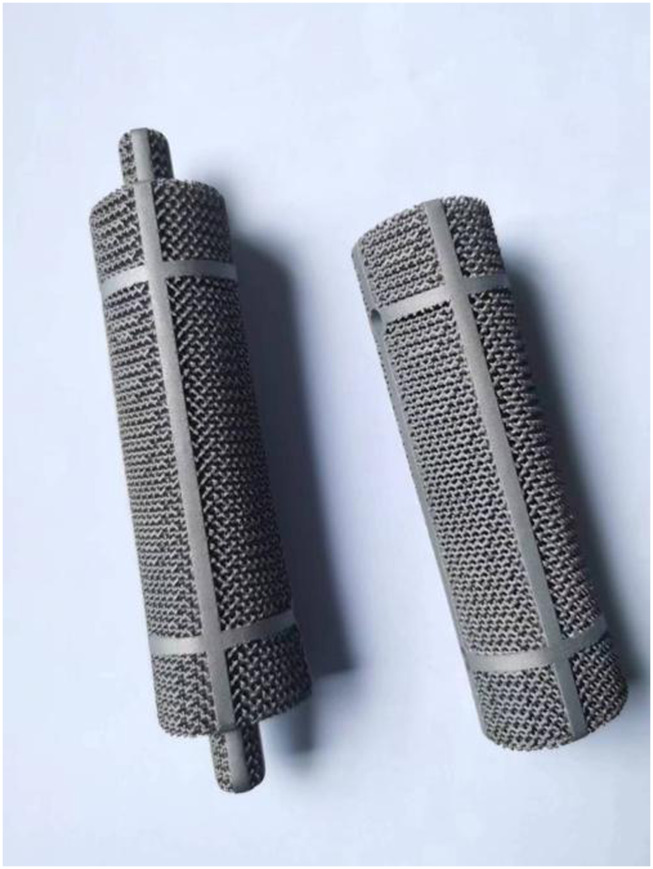
Prosthesis A (left) and B (right).

The use of stiffeners can enhance the stability and load-bearing capacity of the prosthesis, reduce loosening and displacement, and promote bone tissue growth and repair through the combination with a porous structure (Putzer et al., 2024). We designed and manufactured two 3D-printed Ti6Al4V femoral shaft prostheses with the memorable anatomic landmarks and natural skeletal structures from the intact Sawbone. Prosthesis A has a design resembling intramedullary nails. Prostheses B features a hollow structure (Figure 1).

**FIGURE 2:**
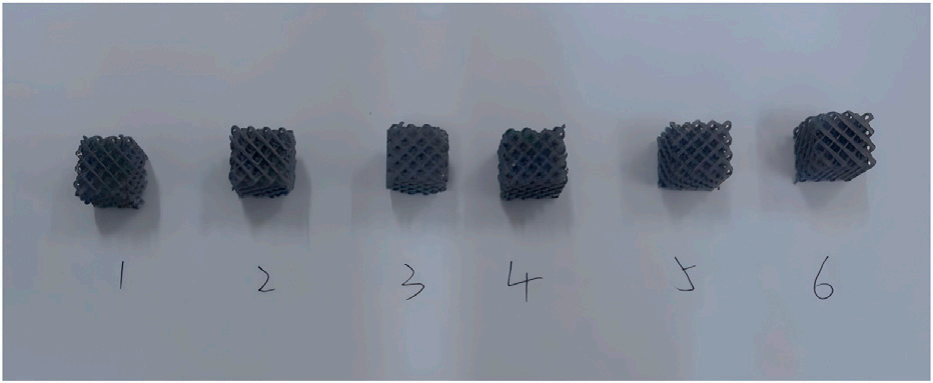
Porous structure samples of prosthesis A and B.

We prepared six samples with the same porous structure as prosthesis A and B. Each one consists of a 4 × 4 × 4 array of cubic unit cells (Figure 2).

**FIGURE 3:**
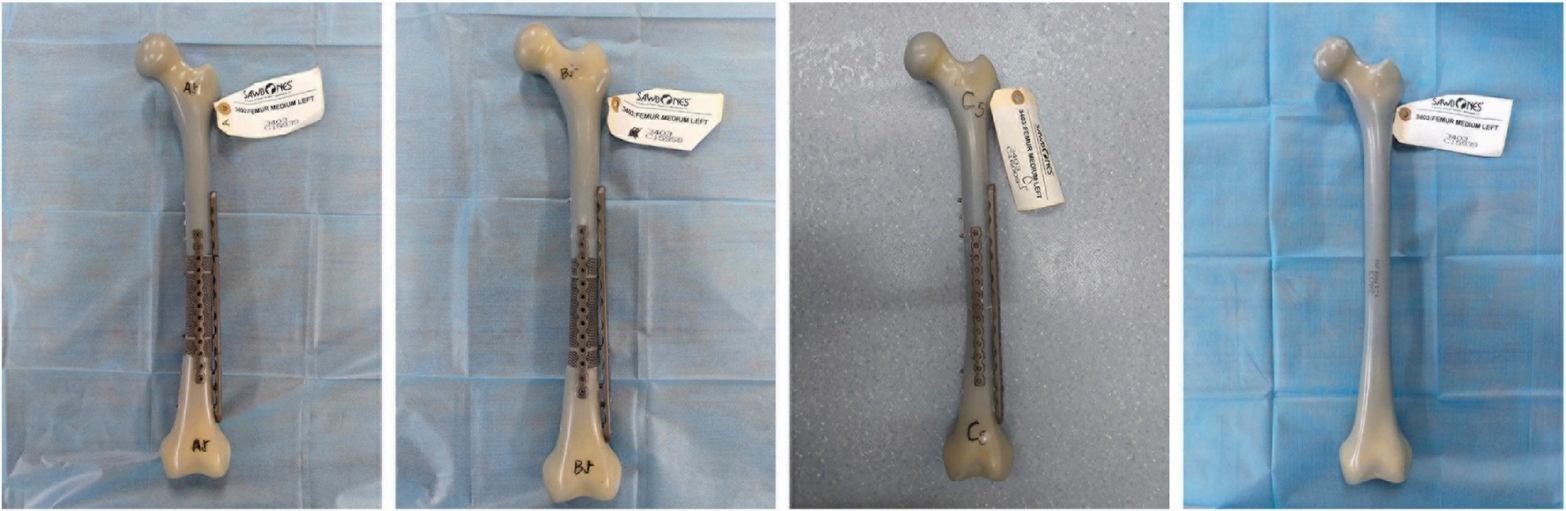
Constructed fracture models from left to right: Group A, Group B, Group C, Group D.

Sixteen fourth-generation Sawbones composite femur (femur medium left, Sawbones3403, Pacific Research Labs, Vashon, WA, United States), screw nail, and plate (XRMED, Suzhou, China), femoral shaft prosthesis and its specimen (LeiHe, Shenzhen, China) were used for the biomechanics test. The composite Sawbones were prepared by an experienced orthopedic surgeon (JJ. Z) and his assistants (HD. L and XY. N). We prepared 10 Sawbones to simulate a 10 cm femoral shaft defect and five Sawbones to simulate a femoral shaft transverse fracture. We use the left intact Sawbone to simulate a normal femur. We divided Sawbones into four groups based on their fixation. Group A: Five femoral shaft defect Sawbones fixed with double plating of prosthesis A. Group B: Five femoral shaft defect Sawbones fixed with double plating of prosthesis B. Group C: femoral shaft transverse fracture fixed with double plates. Group D: one intact Sawbone (Figure 3). In the prosthesis group, the main plate was secured to the femoral segments with three cortical screws at both its proximal and distal ends, and two additional screws were inserted through the plate into the prosthetic stem; the auxiliary plate was fastened to the bone with two screws at each extremity. For the transverse fracture group, the main plate was attached to the femur with three screws at both ends, while the auxiliary plate was fixed by two screws proximally and two screws distally.

### 2.1 Biomechanical tests

**FIGURE 4:**
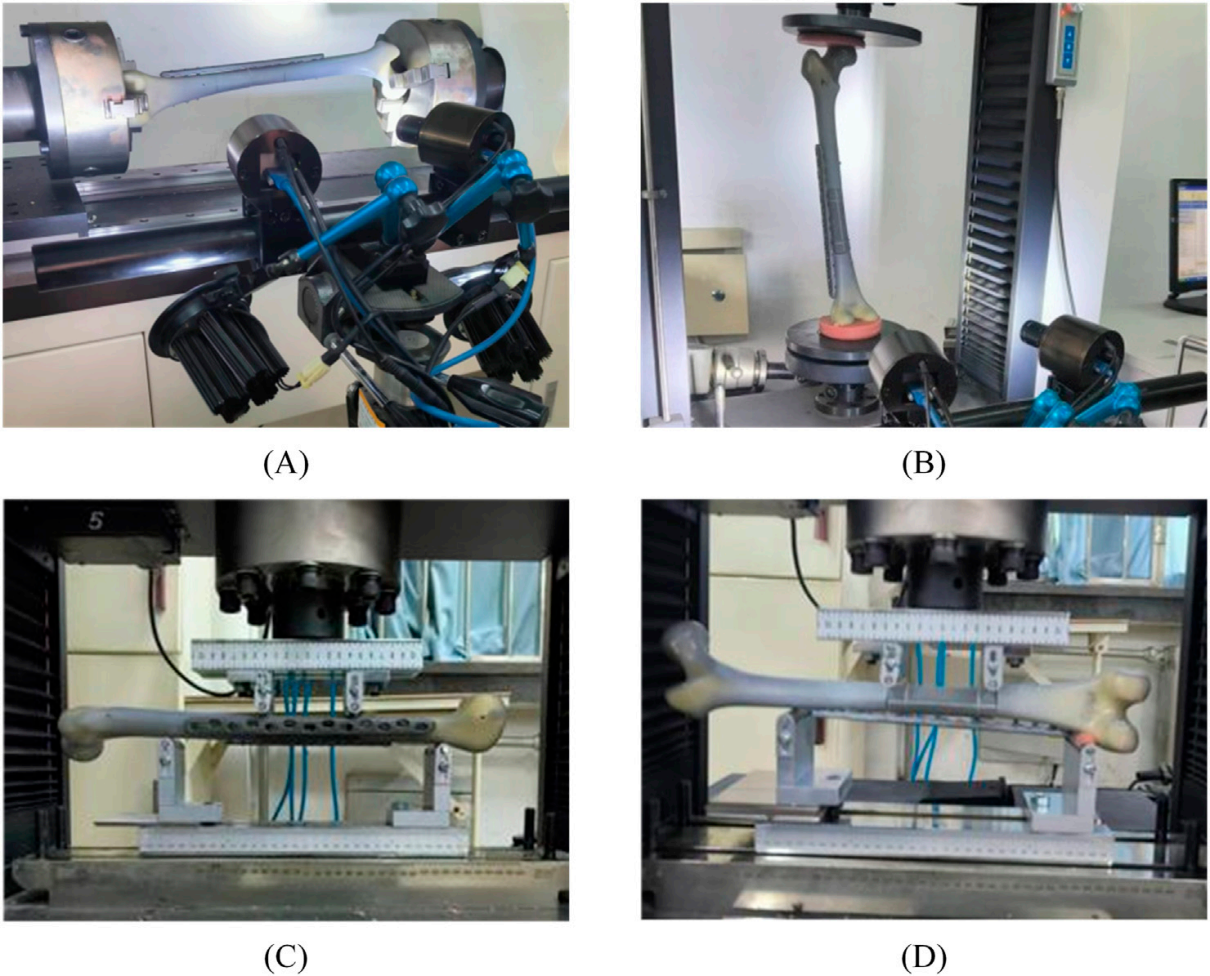
Detailed biomechanical experimental method: **(A)** Sawbone model fixed to torsional test machine, **(B)** Sawbone model fixed to torsional test machine, **(C,D)** Sawbone model fixed to four-point bending test machine. C sagittal section, D coronal section.

Our biomechanical tests were conducted in a rigorous and systematic manner. During the torsion test, the femurs of each group were secured at their proximal and distal ends along the mechanical axis using the testing machine (Figure 4A). The torsional load range was set from −6 Nm to 6 Nm, with a 3°/min loading rate. For the axial compression study, the femurs were secured at both ends using supports fabricated from dental molding powder (Figure 4B). A preload of 10 N was applied, followed by loading at a rate of 20 mm/min up to a maximum load of 870 N—the four-point bending test assessed bending stiffness in the coronal and sagittal sections (Figures 4 C,D). The experiment began with a preload of 10 N, followed by a 1 mm/min loading rate, up to a maximum load of 870 N to simulate standing on one foot (Schneider et al., 2001). All the experiments described above were conducted in five replicates, and the corresponding stiffness results were recorded.

Due to the constraints of DIC technology, direct strain measurement of porous prostheses is not feasible. After the torsion and compression tests, select one femur from groups A, B, and C. The steel plate surface was first coated with white matte spray paint, followed by the random application of black speckle markings. The compression and torsion experiments were repeated to obtain strain measurements of the main plate.

Six porous structure samples were arranged in a 4 × 4 × 4 array and placed on a compression testing machine. A preload of 1 N was applied and automatically zeroed. The platen then uniformly applied pressure to the samples at a 1 mm/min speed until the samples fractured. The effective elastic modulus is calculated using the following formulas. (E is the elastic modulus, ε is the strain, σ is the stress, F is the external load, A is the equivalent cross-sectional area of the sample, X is the original height of the sample, and X1 is the current height of the sample.)

$$E=\frac{\varepsilon }{\sigma }$$

$$\sigma =\frac{F}{A}$$

$$\varepsilon =\frac{X-X_{1}}{X}$$

### 2.2 Finite element analysis

#### 2.2.1 Model conducting

**FIGURE 5:**
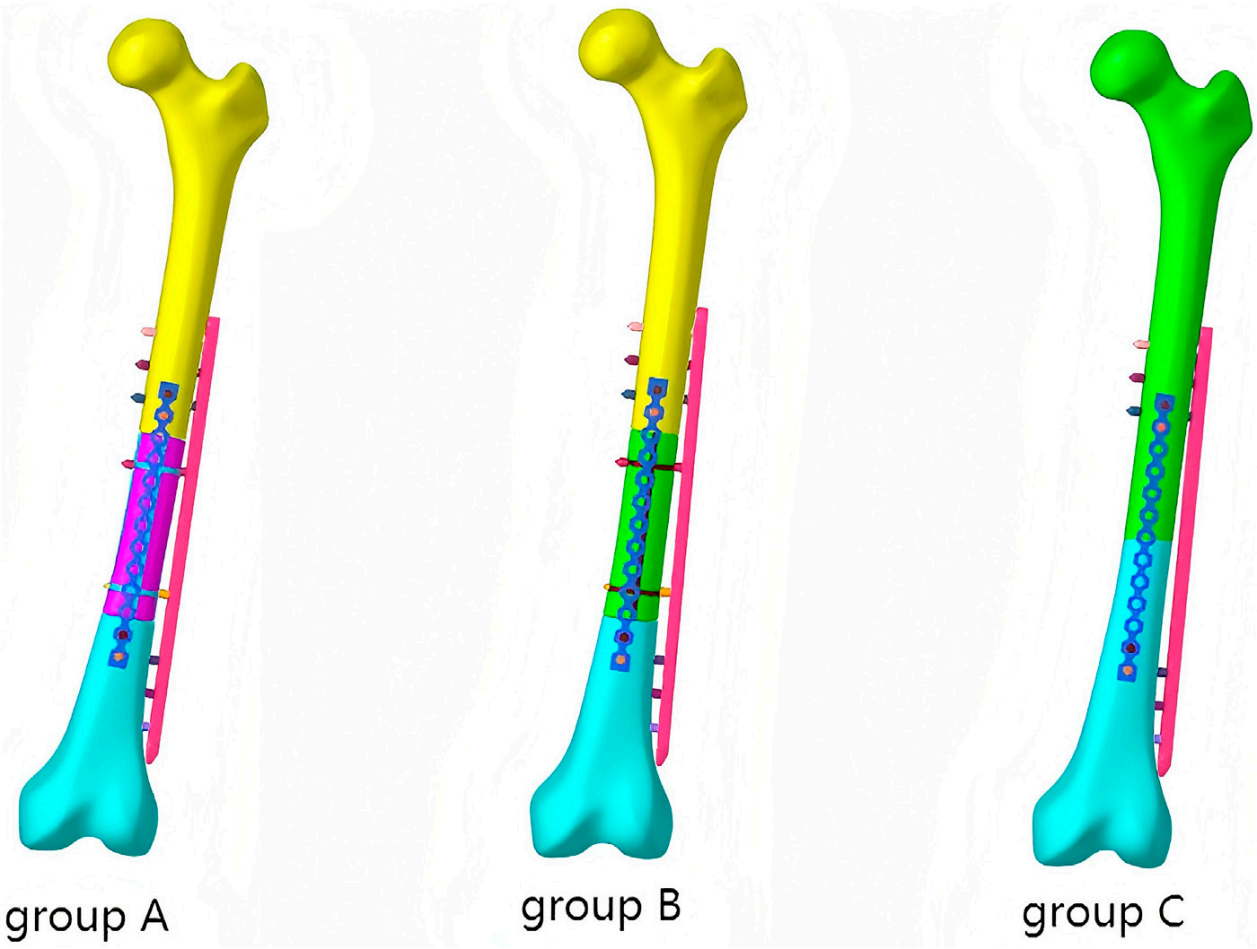
Fracture fixation models of group A, B, and C in finite element analysis.

The CT files of Sawbone were imported into Mimics 21.0, and then 3-Matic was used for the smoothing process. We use Geomagic to construct an intact femur model. Subsequently, we obtained the models of the cortical and cancellous bones through Boolean operations in SolidWorks. The above models were imported into HyperMesh for mesh generation. Then, we conducted the model of femoral shaft defect and femoral shaft transverse fractures based on the intact femur model. In the finite element model, the prosthesis, screws, and plates were assembled onto the femur exactly as in the physical experiment (Figure 5). Each model was characterized by linear elasticity and isotropic behavior, with the modulus of elasticity and Poisson’s ratio provided as reference values (Table 1). The screw-bone, plate, and prosthesis interactions were modeled with tied constraints. The prosthesis-bone and plate-bone interfaces were assumed as surface-to-surface contact relationships with a friction coefficient of 0.2 and 0.15, respectively. In establishing the femoral shaft transverse fracture model, a friction coefficient of 0.2 was used for contact interactions with a 0.1 mm gap between the fracture surfaces.

**TABLE 1:** The material properties and element types.

Component	Young’s modulus (MPa)	Poisson’s ratio	Element type
Cortical	16,700	0.26	C3D10M
Cancellous	155	0.3	C3D10M
Ti6Al4v	114,000	0.34	C3D10M
Poros structures	1,132.85	0.34	C3D10M

#### 2.2.2 Model validity verification

We constrained the distal end of the femoral model and applied the pressure at the coupling point on the femoral head. We compared the result with the previously published literature, showing stress concentration on the medial and lateral sides of the femoral shaft, with the maximum von Mises stress shown below the greater trochanter on the inner side of the femur, similar to the results of the previous study (Mathukumar et al., 2019). The compressive stiffness of the model is 1,309.4 N/mm, which is comparable to the values for the artificial femur (1,263–1317 N/mm), and the cadaveric femur (450–1467 N/mm) from experimental studies (Papini et al., 2007). When a 6 Nm torque is applied at the proximal femoral head, the torsional stiffness of the model is found to be 12.45 Nm/deg, which is similar to the previous study (13.99 Nm/deg) (Amanatullah et al., 2014).

#### 2.2.3 Load application

**FIGURE 6:**
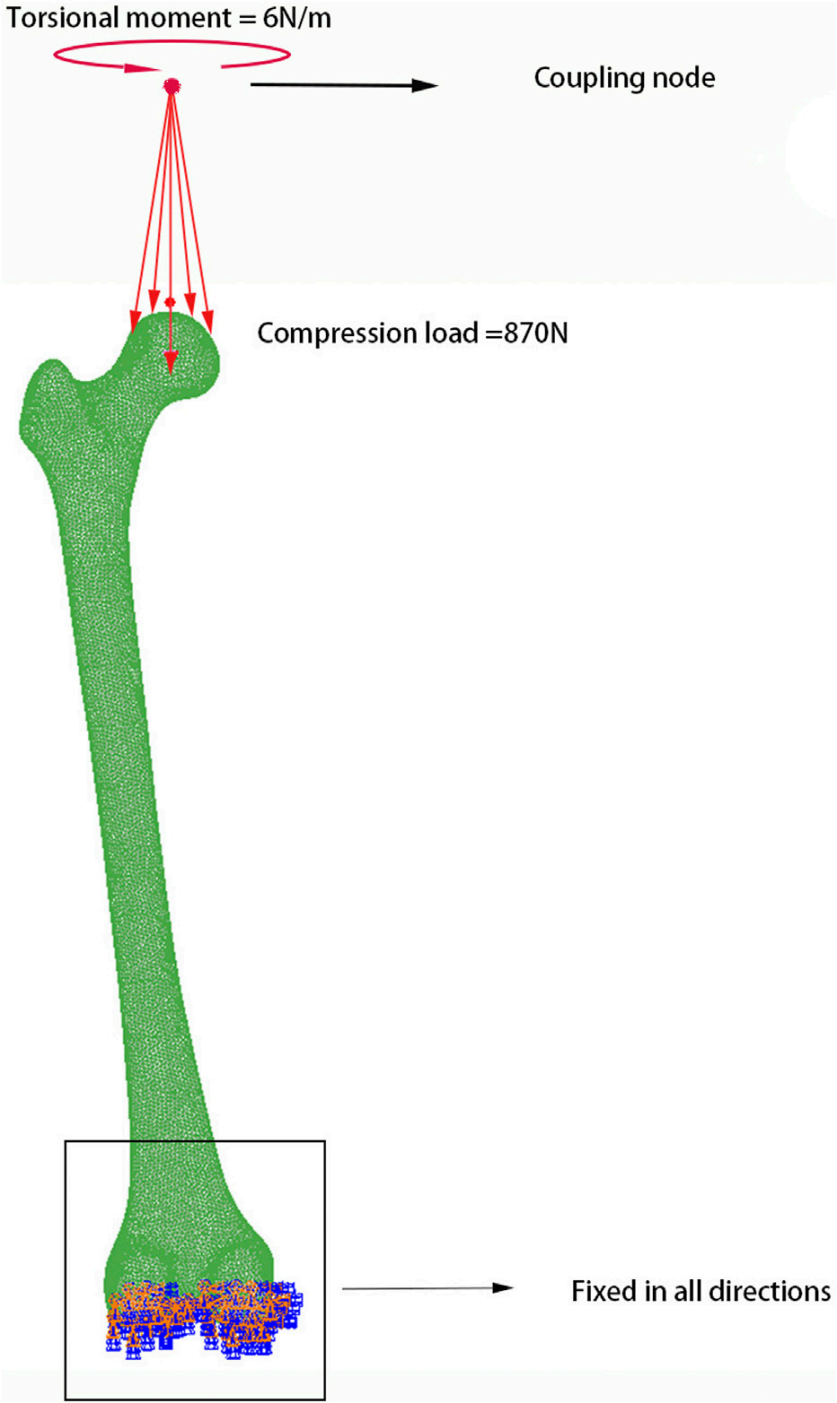
Boundary conditions and load application of the models.

Utilizing the methods from prior investigations (Zhan et al., 2024), the boundary conditions were defined, as illustrated in Figure 6. The distal femur was constrained with a six-degree-of-freedom rigid condition, fixing all nodes in the intercondylar fossa. Loading was applied proximally via a coupling constraint: a concentrated force was exerted at the coupling node above the femoral head and evenly distributed to the relevant mesh elements. In the compression simulation experiment, a force of 870 N was applied at the coupling point. In the torsion simulation experiment, a −6 Nm torque was applied at the coupling point to simulate femoral internal rotation.

### 2.3 Cyclic fatigue and failure tests

The fatigue experiment can simulate the prosthesis’s mechanical behavior during long-term use, assess its fatigue life and durability under repeated loads, and ensure that it maintains good performance during its expected service life.

**FIGURE 7:**
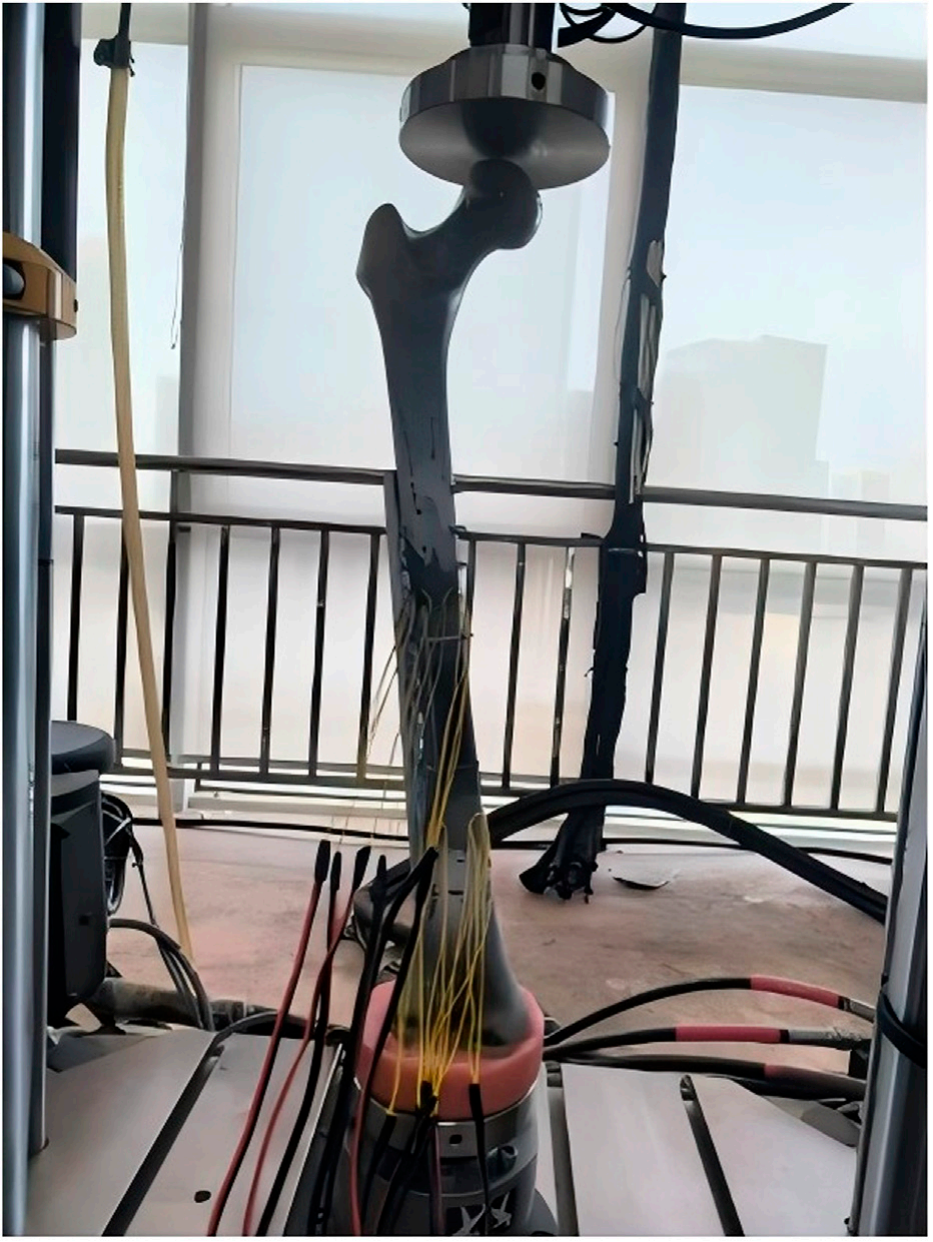
Detailed cyclic fatigue and failure test method: Sawbone model fixed into the test machine.

**FIGURE 8:**
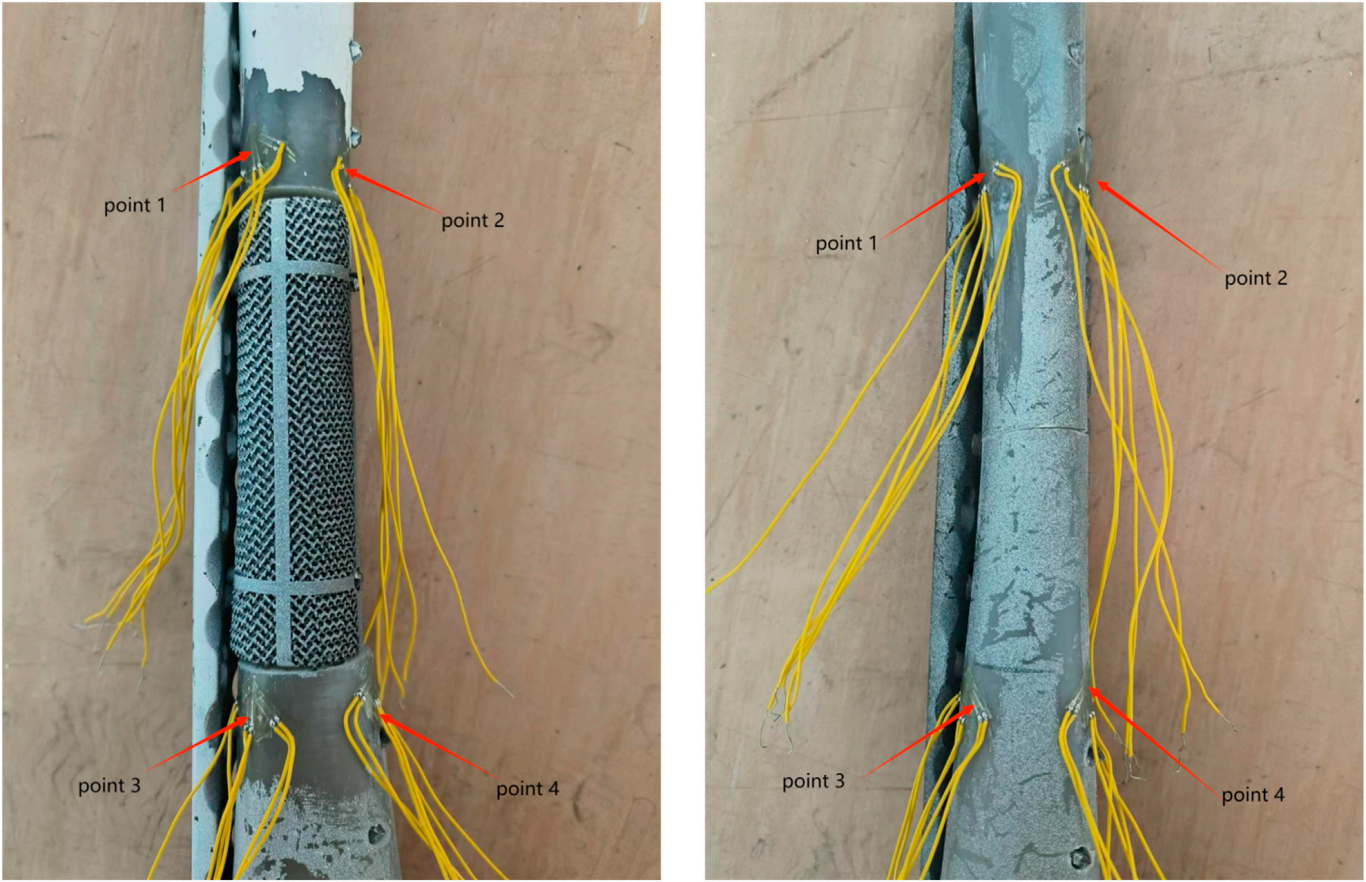
The schematic diagram of the key point fixation. (Left: bone defect group, right: transverse fracture group).

During fixation, femurs were adducted by 15° in the coronal plane, while remaining vertical in the sagittal plane, with the internal rotation angle between 5° and 10°. The femurs were placed on the testing machine to align the mechanical axis during force application (Figure 7). Four three-element strain gauges were fixed on the medial and lateral surfaces of each femur, positioned 210 mm and 230 mm from the proximal end along the anatomical axis, respectively (Figure 8).

The entire fatigue test was conducted in six steps of cyclic fatigue. The specimens then underwent cyclic fatigue loading for 100,000 cycles at 2 cycles per second. Five sets of 20,000 cycles were performed at the following compressive forces: (i) 400 N–1000 N, (ii) 400 N–1200 N, (iii) 400 N–1400 N, (iv) 400 N–1600 N, and (v) 400 N–1800 N. Strain gauges were adhered before the last 10,000 loading cycles.

Lastly, the specimens underwent cyclic loading to failure along the anatomic axis of the femur, starting with 50 cycles of axial loading from 400 N to 1800 N, followed by a 10 s pause at a constant baseline force of 1100 N. The peak force was then increased by 100 N for each subsequent 10 cycles until visible cracks appeared or the stress value dropped significantly.

### 2.4 Statistical data process

This study’s data analysis was carried out using IBM SPSS Statistics 27.0. Owing to the particularity of manually constructed models and the limited sample size, we adopted non-parametric tests to evaluate the differences between groups, with a significance level of 0.05.

## 3 Results

### 3.1 Stiffness

**FIGURE 9:**
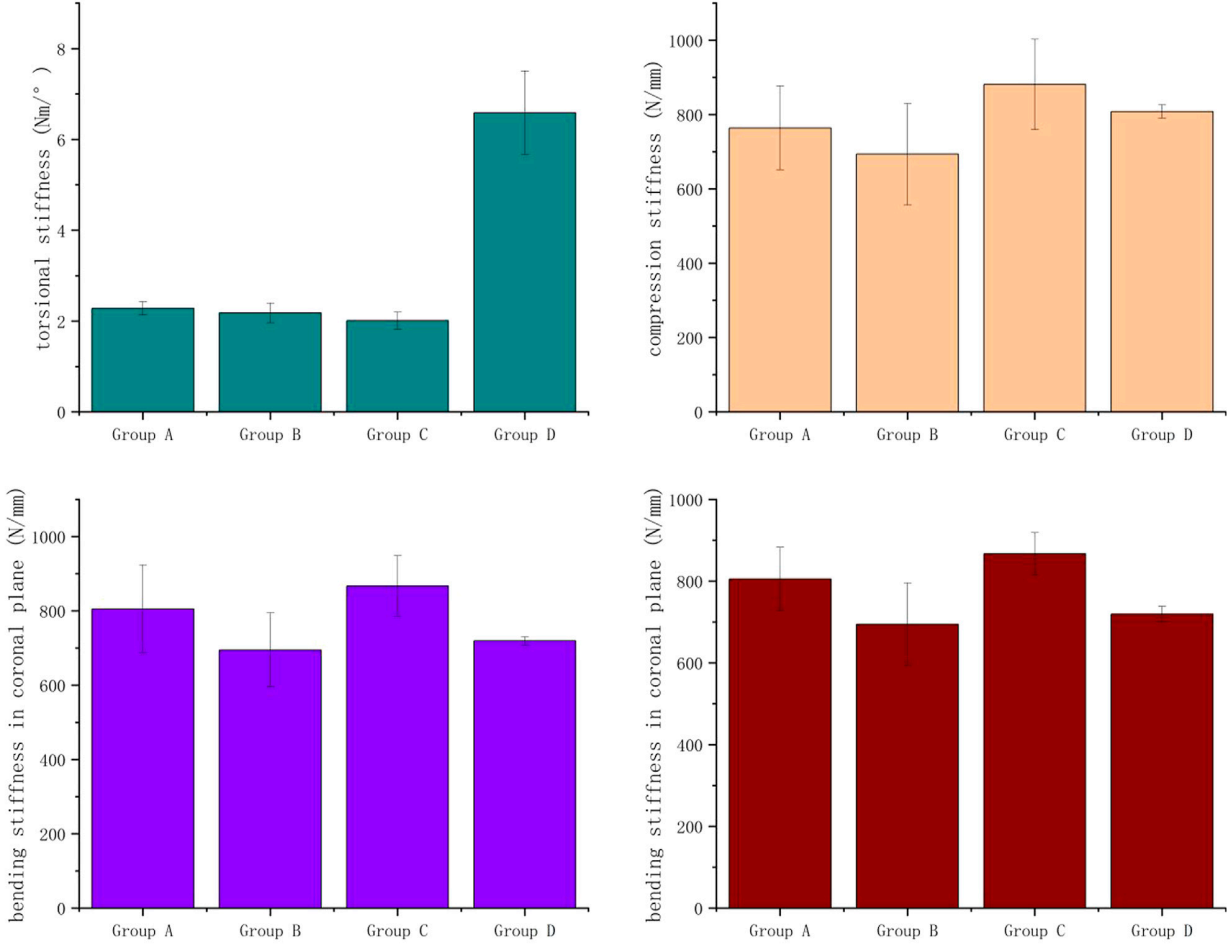
The result of stiffness.

Stiffness is a critical biomechanical factor in assessing the stability and reliability of the prosthesis. Figure 9 shows the results of compression, torsional, and bending stiffness. No significant difference in compression stiffness and coronal bending stiffness was observed between Groups A, B, and D (P > 0.05). The torsional stiffness of Group D is approximately three times that of Groups A, B, and C. No significant difference in stiffness was observed between Groups A and B, except for the coronal bending stiffness.

The average effective modulus of the porous section of prosthesis A and B was 1,132.85 MPa. The elastic modulus lies between the ranges of cortical cancellous bone.

### 3.2 Stress and strain

**FIGURE 10:**
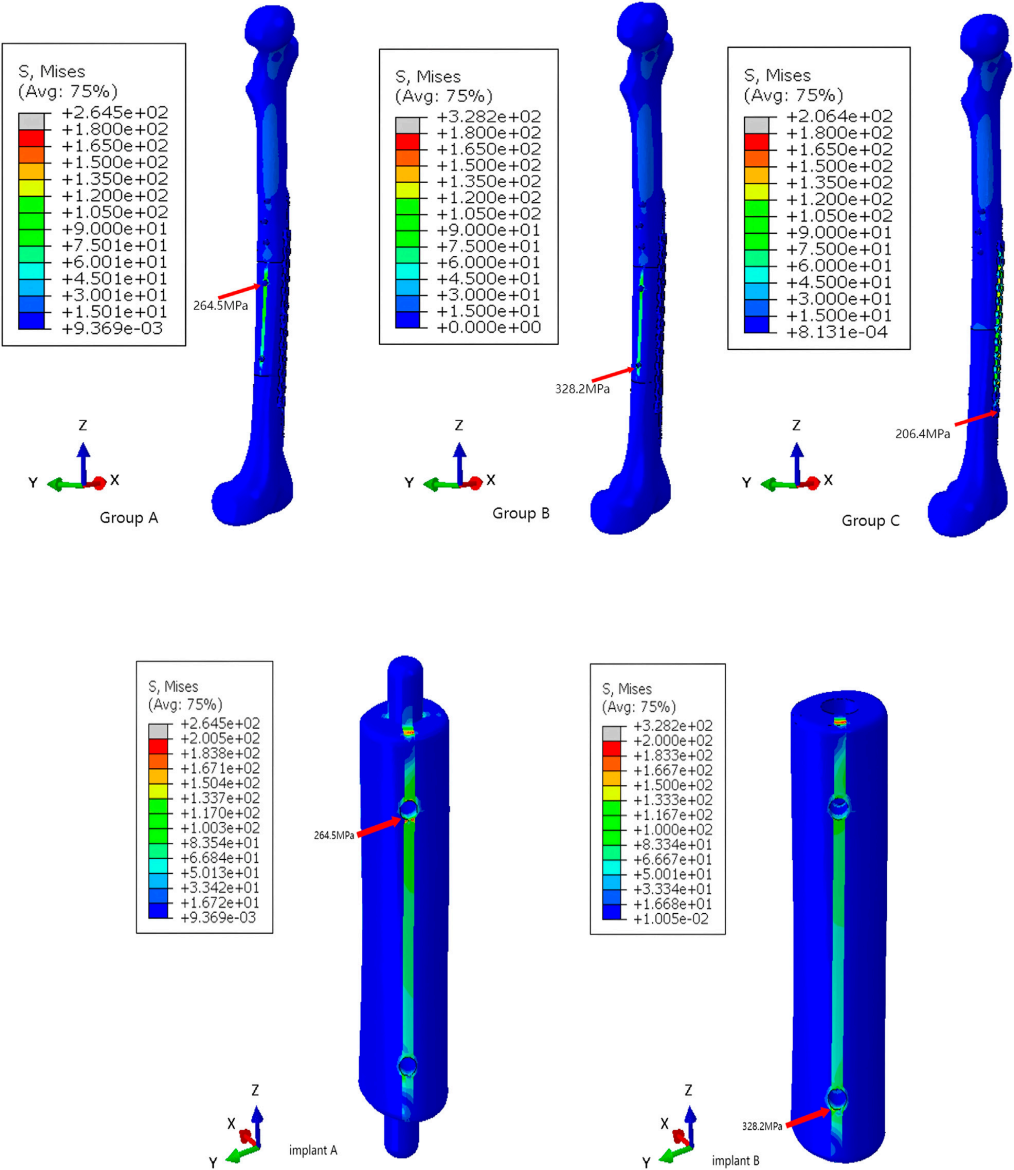
The von Mises stresses distribution within the models and prosthesis under compression loading.

**FIGURE 11:**
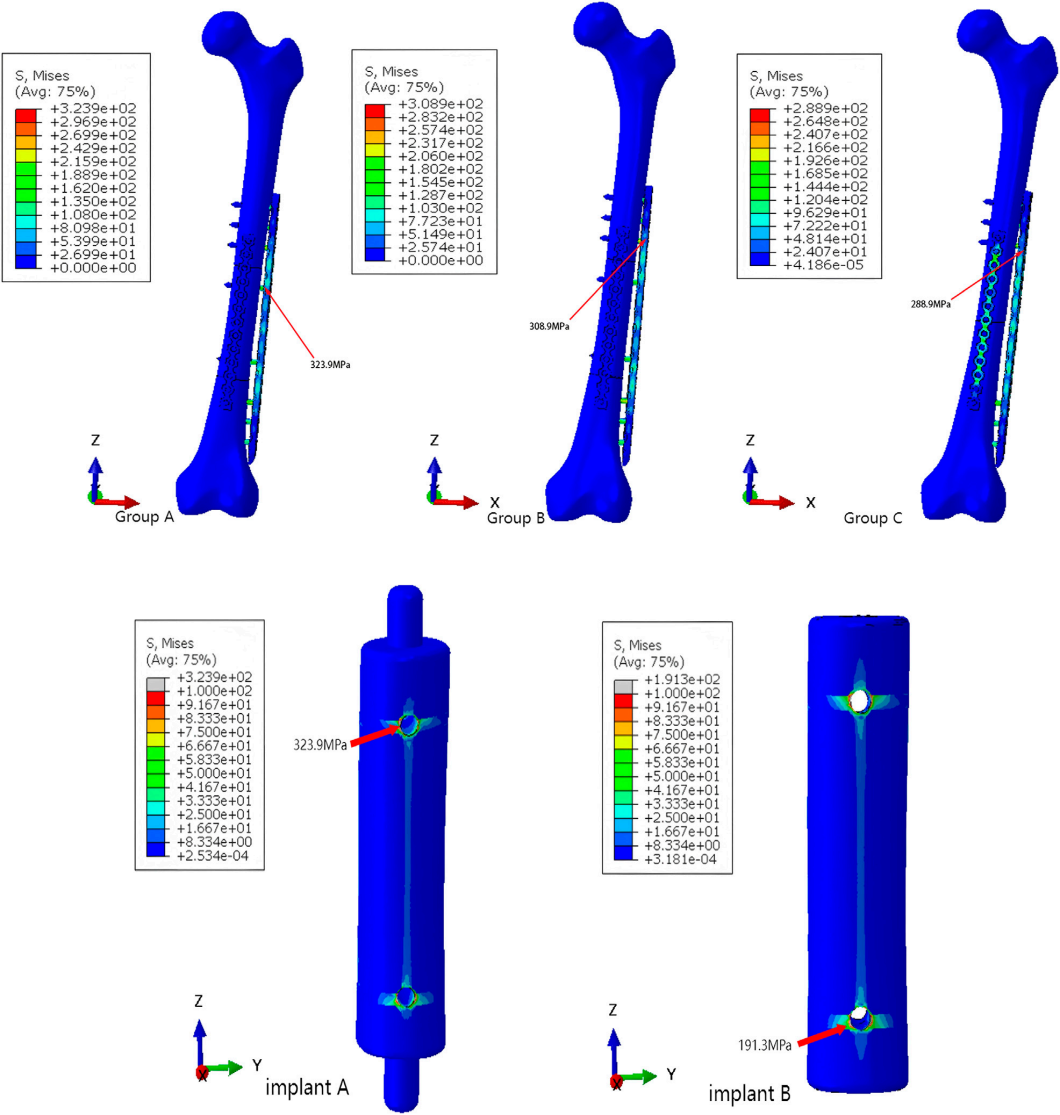
The von Mises stresses distribution within the models and prosthesis under torsional loading.

Based on the stress and strain distribution within the model, we can identify the potential failure under different loading conditions. Under compression load simulation, the maximum von Mises stresses of Group A were 264.5 GPa, the maximum von Mises stresses of Group B were 328.2 GPa, and the maximum von Mises stresses of Group C were 206.4 GPa (Figure 10). Under torsional load simulation, the maximum von Mises stresses of Group A were 323.9 GPa, the maximum von Mises stresses of Group B were 308.9 GPa, and the maximum von Mises stresses of Group C were 288.9 GPa (Figure 11). In Group A, the maximm von Mises stresses occurred at the upper screw hole of the prosthesis. The maximum von Mises stresses in Group B occurred at the lower screw hole of the prosthesis under compression load simulation and the tenth screw hole of the main plate under compression load simulation. The maximum von Mises stresses in Group C were observed at the primary and supplemental plates. We can tell that all groups’ maximum von Mises stresses are lower than the yield strength of titanium alloy (819–1,022 MPa).

**FIGURE 12:**
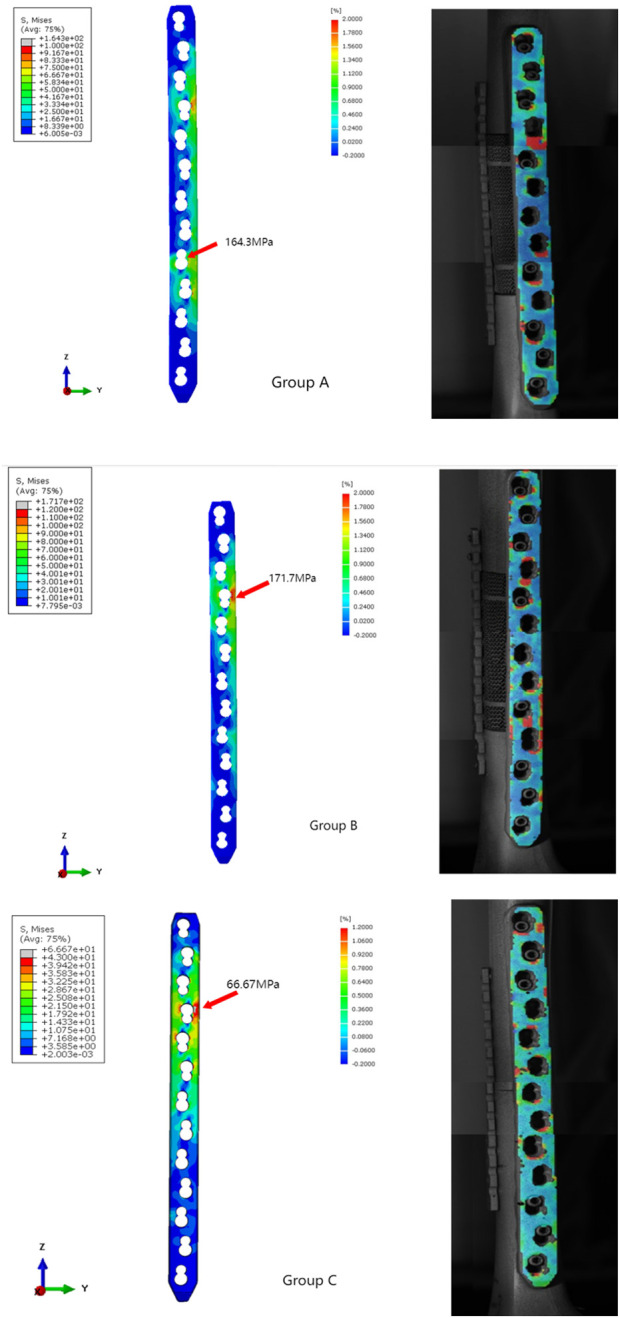
The stress and strain distribution comparison of the main plate under compression load.

**FIGURE 13:**
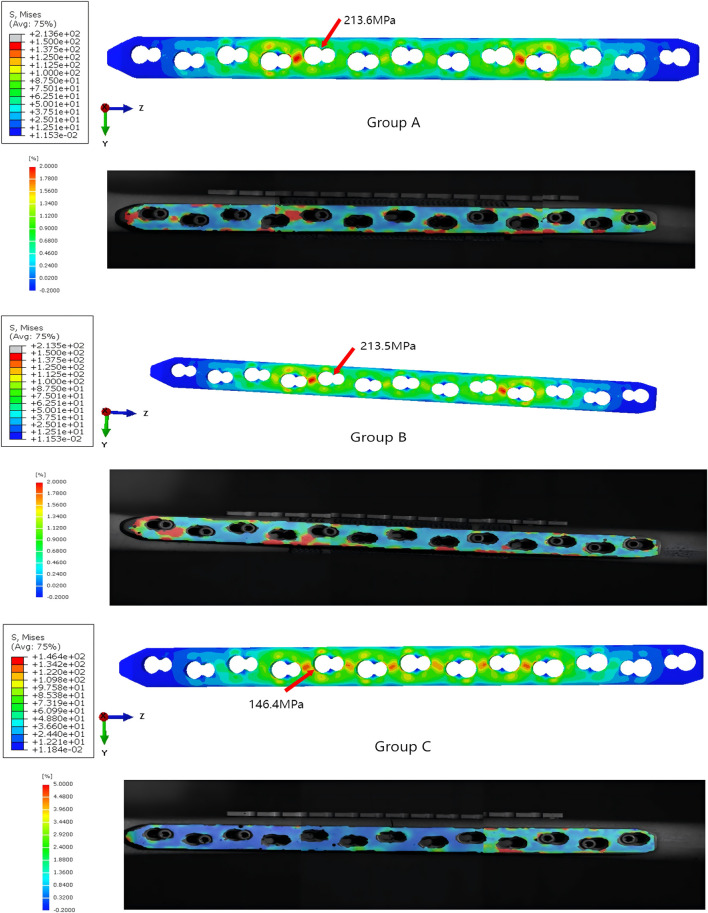
The stress and strain distribution comparison of the main plate under torsional load.

By comparing the stress results from the finite element analysis with the strain results from DIC (Figures 12, 13), we can tell that the strain distribution from DIC closely resembles the stress distribution from the finite element analysis, further validating the effectiveness of the finite element model. It can be seen that both stress and strain are primarily concentrated around the fourth and tenth screw holes of the steel plate. The remaining areas of the steel plate exhibit a uniform distribution, with the maximum stress and strain remaining below the titanium alloy’s yield strength and strain limit.

### 3.3 Displacement amplitude and ultimate load

Tables 2, 3 present the maximum displacement amplitude of each group during cyclic fatigue and failure tests. We also quantified and documented the displacement amplitude variations across all groups before and after the final 10,000 cycles under 400–1800 N loading (Table 4). We can find that the result of displacement amplitude has a substantially high coefficient of variation (CV), which significantly compromises statistical power and increases the risk of Type II errors.

**TABLE 2:** The key point of the displacement amplitude in the cyclic fatigue test (με).

micro-strain (με)	Point 1	Point 2	Point 3	Point 4
Group A	209.55 ± 33.29	698.54 ± 501.57	46.68 ± 29.95	212.24 ± 212.35
Group B	195.32 ± 109.86	571.64 ± 377.82	228.09 ± 116.36	149.18 ± 151.73
Group C	236.40 ± 155.86	648.04 ± 228.90	420.00 ± 159.73	78.71 ± 39.83

**TABLE 3:** The key point of the displacement amplitude in the cyclic failure test (με).

micro-strain (με)	Point 1	Point 2	Point 3	Point 4
Group A	572.43 ± 187.39	2,293.12 ± 1,106.19	244.61 ± 282.11	490.18 ± 415.43
Group B	510.26 ± 166.83	1,193.37 ± 644.92	616.35 ± 514.70	434.35 ± 328.15
Group C	664.78 ± 567.03	2,200.43 ± 397.98	1,407.21 ± 319.63	170.16 ± 83.47

**TABLE 4:** The displacement difference in the last 10,000 cycles (με).

micro-strain (με)	Displacement difference
Group A	38.07 ± 28.06
Group B	30.73 ± 16.88
Group C	12.78 ± 6.30

**FIGURE 14:**
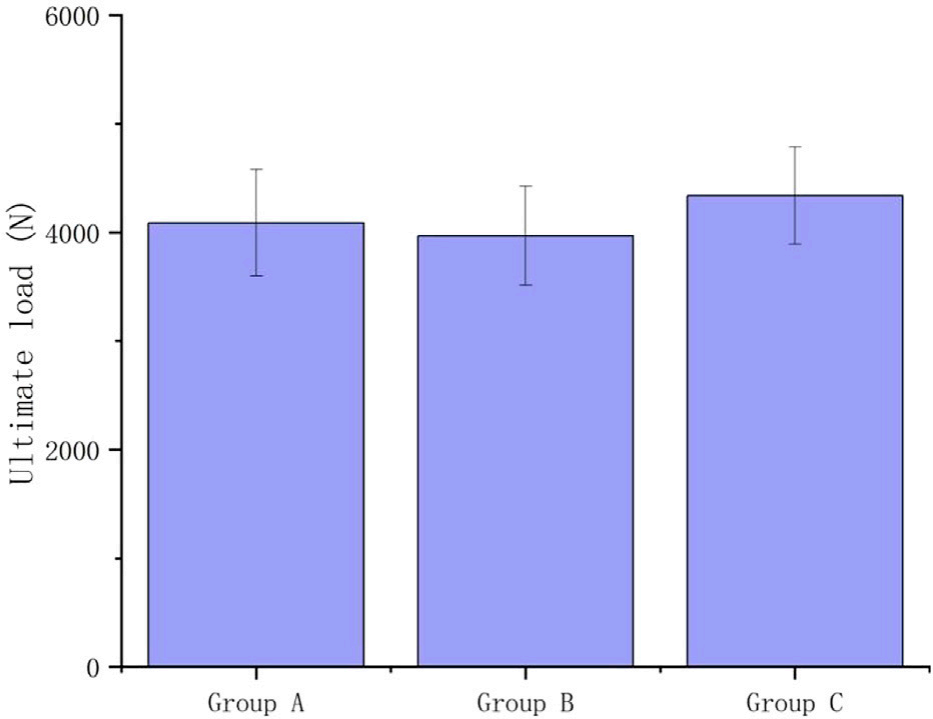
The ultimate load under cyclic failure tests.

From the result of the ultimate load (Figure 14), we can tell that there is no significant difference in ultimate load between Groups A, B, and C (P > 0.05). In Group A, four femurs exhibited failure between the proximal femur and the first screw, while one femur failed at the femoral neck. In Group B, all failures occurred between the proximal femur and the first screw. In Group C, two femurs failed between the proximal femur and the first screw, while the remaining three failed at the femoral neck.

## 4 Discussion

Significant segmental defects of the femoral stem are currently a challenging clinical problem. They are also more likely to occur in the high-explosive-factor environment of modern warfare, where patients require strong prosthetic support to aid in early postoperative mobility, which is particularly important in the battlefield environment (Lawry et al., 2025). The high strength and corrosion resistance of titanium alloy (Ti6Al4V) provide inherent advantages in manufacturing personalized load-bearing bone prosthesis. This material has been used to manufacture a wide range of orthopedic prostheses. However, it has not yet been applied clinically to femoral shaft prosthesis. Through a combination of physical experiments and digital simulations, we aim to systematically evaluate the capacity of prosthetics made from this material to mitigate the biomechanical deficits induced by bone defects.

Through the combination of stiffener and porous structure, we are able to achieve a porosity of more than 70% in the porous part of the structure, while the stiffener maintains the overall rigidity and assists in the transmission of forces.In order to verify the actual growth-in under this theory, *in vivo* animal implantation experiments are underway.

Since our research concentrates on early postoperative exercise and rehabilitation, we avoided strenuous activities like running and jumping and instead opted for simulated steady walking. Despite these limitations, the importance of early postoperative mobility and gait restoration in military rehabilitation cannot be overstated, especially for high-energy lower limb injuries sustained in battlefield settings. Early mobilization has been shown to enhance fracture healing, reduce postoperative complications, and improve overall patient outcomes in trauma scenarios.

Groups A, B, and C mimic the unhealed model for the significant difference with Group D in torsion stiffness. The torque was transmitted through the screws to the plate, thereby restricting the torsional displacement of the proximal and distal femoral segments. The presence of bone discontinuity and gaps leads to an increased relative displacement between the proximal and distal femoral segments, which explains why the torsional stiffness of the normal femur is markedly higher than that of the other three groups. Compared to Group C, Groups A and B demonstrated more significant gaps between the proximal and distal fracture segments. Two extra screws linking the prosthesis with the central plane led to minimal variation in torsional stiffness across Groups A, B, and C.

No statistically significant differences were observed in compressive stiffness between groups A, B, and D during the compression experiment. Group C exhibited a marginally higher compressive stiffness than the other three groups, which can be attributed to the steel plate’s greater rigidity. The similarity in compressive stiffness between groups A, B, and D is partly due to the prosthesis’s low elastic modulus, which approximates cancellous bone (Liu et al., 2013). The effective elastic modulus close to cancellous bone reduces the stress shielding effect and improves the prosthesis’s mechanical compatibility with the bone tissue (Didier et al., 2017). The material’s elastic modulus reduction arises from a suitably designed porous structure. The results demonstrate that, in macro-scale biomechanical experiments, prosthesis A and B can effectively compensate for the biomechanical disadvantages of the femoral shaft defect.

The maximum von Mises stresses is a critical parameter for assessing the structural integrity of prostheses. When the maximum von Mises stresses surpasses the yield strength of the prosthesis, it will deform or potentially fracture. The maximum stress and strain obtained from the finite element analysis and DIC experiment were both below the titanium’s ultimate strength, indicating that the component possesses sufficient strength reserves and is capable of safely withstanding the applied loads.

High CV was observed in the result of displacement amplitude, with CV in some groups over 80%. We speculate that two potential factors are responsible for the result. First, the manual preparation of fracture models without an osteotomy template introduced slight angular deviations in the osteotomy surfaces. This could significantly affect the stress and strain distribution at key points, such as points 1 and 2. Clinically, these differences can be mitigated through bone-graft gap filling or customized osteotomy guides to optimize the osteotomy surface. Second, examination of the samples post-testing identified considerable bone debris on their surfaces and inside the prosthesis, resulting from the numerous fatigue cycles applied in a short time. This debris, produced by wear on the osteotomy surfaces, exacerbated inter-group differences, further increasing the CV in the results.

No notable deformation was observed under 100,000 loading cycles, equivalent to 13.3 years of walking for a normal adult. This indicates the product’s potential for long-term durability in supporting daily loads. In Group A and Group B, the ultimate load approached 4000 N, approximately six times the typical human body weight, with no failure in the prosthesis system with dual steel plates. These findings suggest that prosthesis A and B can withstand various unexpected impact forces, even during the early post-surgery stages.

No load drops in these plots are associated with the crack pop-in events observed in the prosthesis during the cyclic fatigue and failure tests. A comparison of the fracture locations among the three groups showed that no new fracture-prone areas were introduced in Groups A and B. However, fractures were more likely to occur at the connection between the proximal femur and the first locking screw in Groups A and B. This may be attributed to the large deformation induced by the fracture gaps in Groups A and B, which is transmitted through the plate and screws to the upper femoral shaft. We suggest that in clinical applications, the gap can be filled with bone fragments, and the issue can be significantly improved as the bone grows into the porous structure of the prosthesis, thereby eliminating the gap between the prosthesis and the femur.

## 5 Conclusion

In modern warfare, the types of casualties are different from previous wars, which shows that various types of remotely dropped explosive devices are the hallmark threat of current military operations. Treating open fractures and bone defects caused by blast injuries is a complex issue in clinical orthopedics (Xue et al., 2022). The inherent properties of titanium alloys make them an ideal material for the construction of prostheses intended to replace load-bearing bones like the tibia and femur. Prostheses A and B effectively compensate for biomechanical disadvantages in segmental femoral defects, matching the performance of intact femurs. Their design ensures uniform stress distribution, safe stress and strain levels, durability under cyclic loading, and preservation of natural load transmission.

The distinct characteristics of prostheses A and B offer clinicians expanded options. Prosthesis A is characterized by superior stability and less requirements for the surgeon’s experience. The hollow design of prosthesis B enables a greater variety of filling materials. Because the prosthesis eliminates the time for bone growth and directly replaces the site of the bone defect, titanium prosthetic repair allows for immediate stabilization, rapid recovery, and early weight bearing, which not only meets the current clinical requirements for surgical treatment of critical bone defects, but also meets the requirements in the battlefield. As long as a suitable bone-soft tissue operative interface can be reconstructed preoperatively, the 3D-printed titanium alloy prosthesis combined with a dual-plate fixation system developed in this study can be effectively and securely used for a wider range of non-combat injuries, including those from high-energy motor vehicle collisions and post-tumor excision defects. The system’s biomechanical compatibility, ease of use in surgery, and modular design collectively support its progress toward clinical application. While our findings highlight the prosthesis’s effectiveness in aiding early activities, they also support broader evidence indicating that such basic mobility is essential for ultimately achieving combat-relevant performance. Promoting the use of the prostheses can increase casualty survival rates, improve the ability to treat combat injuries and ensure and improve the combat effectiveness of troops.

## Data Availability

The original contributions presented in the study are included in the article/supplementary material, further inquiries can be directed to the corresponding authors.
