# Correction: Biomechanically optimized 3D-printed titanium prostheses with stiffener arrangement for critical femoral diaphyseal defects: early weight-bearing capacity and combat readiness validated through integrated biomechanical-FEA approach

**DOI:** 10.3389/fbioe.2025.1709142

**Published:** 2025-10-27

**Authors:** Guo-Sen Li, Hao Li, Da Liu, Rui Yi, Yi Cui, Hong-Da Lao, Xiao-Yang Nie, Min Zhao, Cheng-Fei Du, Yong-Qing Xu, Jiang-Jun Zhou

**Affiliations:** ^1^ Department of Orthopaedics, The 908th Hospital of Joint Logistic Support Force, Nanchang, Jiangxi, China; ^2^ Third Clinical Medical College of Nanchang University, Jiangxi Medical College, Nanchang University, Nanchang, Jiangxi, China; ^3^ The General Hospital of Western Theater Command, Chengdu, Sichuan, China; ^4^ The Seventh Medical Center, Chinese PLA General Hospital, Beijing, China; ^5^ Institute of Orthopedic Trauma, 920th Hospital of Chinese PLA Joint Logistics Support Force, Kunming, Yunnan, China; ^6^ Department of Orthopaedics, The People’s Hospital of Yingtan City, Yingtan, Jiangxi, China; ^7^ Tianjin Key Laboratory for Advanced Mechatronic System Design and Intelligent Control, School of Mechanical Engineering, Tianjin University of Technology, Tianjin, China

**Keywords:** 3D-printed prosthesis, critical bone defect, finite element analysis, biomechanical compatibility, military trauma, stiffener, titanium alloy

Author Hao Li was erroneously assigned as **corresponding author**. The correct corresponding authors are Cheng-Fei Du, Yong-Qing Xu, Jiang-Jun Zhou.

There was a mistake in Figure 3 as published. Figure 3 in the PDF is not as sharp as we originally expected, and group C in the previous Figure 3 was inadvertently shown before the assembly was complete. To remove any potential ambiguity or misinterpretation we have now prepared a fully assembled, higher-resolution image. The corrected Figure 3 appears below.

**FIGURE 3:**
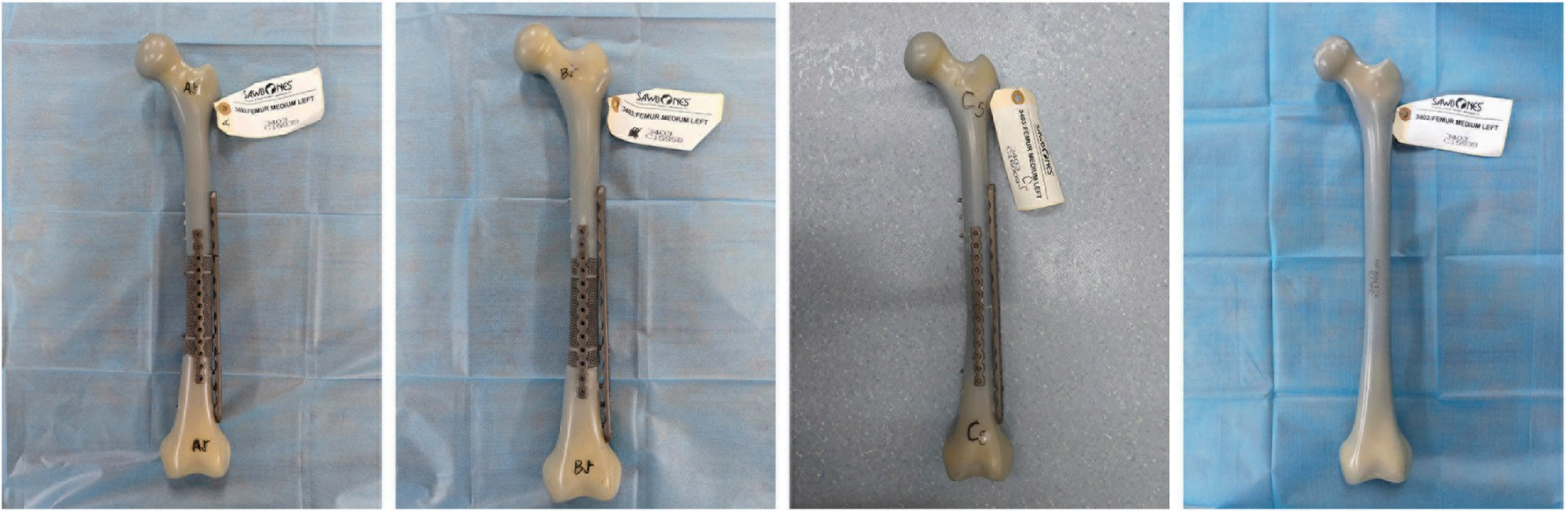
Constructed fracture models from left to right: Group A, Group B, Group C, Group D.

The original article has been updated.

